# Association between awareness of government notification and ‘negative hepatitis virus test result card’ and implementation of hepatitis B and C screening in Japanese workplaces: a cross-sectional study

**DOI:** 10.1186/s12889-026-28932-7

**Published:** 2026-08-07

**Authors:** Shoko Nakazawa, Kosuke Sakai, Kota Fukai, Yuko Furuya, Kei Sano, Masayuki Tatemichi, Masaaki Korenaga

**Affiliations:** 1 Department of Preventive Medicine, Tokai University School of Medicine, 143 Shimokasuya, Isehara City, Kanagawa 259-1193 Japan; 2 Department of Ophthalmology, The Jikei University School of Medicine, Tokyo, Japan; 3 Hepatitis Information Center, The Research Center for Hepatitis and Immunology, National Institute of Global Health and Medicine, Japan Institute for Health Security, Ichikawa, Japan

**Keywords:** Hepatitis B, Hepatitis C, Negative hepatitis virus tests result cards, Public health, Policy

## Abstract

**Background:**

‘Request for cooperation on measures against viral hepatitis in the workplace’ (the notification) was issued to provide hepatitis screening opportunities in workplaces. However, employees may take hepatitis tests multiple times because the results are not recognised. ‘Negative hepatitis virus test result cards’ (NHVC), designed to certify negative results for hepatitis B virus (HBV) and hepatitis C virus (HCV) tests, were developed to decrease multiple screenings. We aimed to examine the association between word awareness of the notification/NHVC, and hepatitis testing opportunities provided by employers, as well as the knowledge regarding hepatitis and the presence of employees with HBV/HCV infection.

**Methods:**

An online questionnaire survey was conducted in Japan in March 2024 among executives and human resources personnel. Having or lacking word awareness of the notification/NHVC was defined as responding as ‘know about it/heard of it’ or ‘never heard of it’ to these items. The number of employees with opportunities for hepatitis testing, number of employees with HBV/HCV, and number of correct answers in hepatitis quizzes were assessed. Odds ratios (ORs) and 95% confidence intervals (CIs) were estimated using logistic regression to examine associations of word awareness of the notification/NHVC with providing hepatitis testing and hepatitis quiz answers, while examining the association of employees with HBV/HCV with word awareness of the notification/NHVC.

**Results:**

Respondents (*n* = 1,500) with word awareness of the notification/NHVC had higher odds of providing hepatitis testing opportunities (notification: OR: 9.63, 95% CI: 7.34–12.6 and NHVC: 10.8, 8.12–14.4) and correct responses (OR: 1.87, 95% CI: 1.49–2.34 and 1.45, 1.15–1.83, respectively) compared to those who were unaware, after adjusting for occupation (executives/human resources personnel) and company size. Respondents with employees with HBV/HCV were more likely to be word-aware of the notification /NHVC than those without such employees (OR: 6.42, 95% CI: 4.62–8.93 and 4.9, 3.59–6.68, respectively).

**Conclusions:**

Word awareness of the notification/NHVC was positively associated with hepatitis testing, correct responses, and the presence of employees with HBV/HCV in companies. It also aided in identifying workplaces with greater engagement in hepatitis testing. Future studies should investigate barriers to hepatitis screening and relevant interventions.

**Supplementary Information:**

The online version contains supplementary material available at https://doi.org/10.1186/s12889-026-28932-7.

## Background

Although the World Health Organization set the goal of eliminating hepatitis caused by hepatitis B virus (HBV) and hepatitis C virus (HCV) by 2030 [1], Cui et al. estimate 89.7% and 78.6% of respective infections remain undiagnosed worldwide [2]. In Japan, although HBV/HCV incidence is declining, the number of people with the respective infections were estimated at 1.02 − 1.19 million and 0.87 − 1.3 million in 2015 [3]. Meanwhile, the estimated burden of undiagnosed and untreated disease has been projected to reach approximately 0.44 − 0.56 million and 0.92 − 3.57 million by 2030 for HBV and HCV, respectively [3]. Japan has implemented comprehensive hepatitis control for HBV and HCV under the Basic Act on Hepatitis Control. Municipalities screened 10 million people under the urgent hepatitis virus testing service from 2010 to 2017 [4]; however, Tatemichi et al. found in 2016 that among 10.5 million workers, only 5.23% (0.49 million) of individuals had been screened for HBV and 3.82% (0.31 million) for HCV in workplace [5]. In March 2023, the Ministry of Health, Labour and Welfare of Japan issued the notification ‘Request for cooperation on measures against viral hepatitis in the workplace’ (hereafter referred to as the notification) [6] to employers to implement HBV/HCV screening at workplaces. The notification described five aspects: informing workers about the significance of hepatitis virus testing, providing testing opportunities, ensuring privacy, supporting the balance of treatment and work, and preventing discrimination [6]. Occupational health and safety (OHS) policies are sometimes announced to promote health awareness, and this notification did not include penalties [7].

Participating in hepatitis screening in the workplace can be convenient for employees who undergo mandatory annual health checks in Japan [8]. However, some employees may undergo multiple screening tests because, although test results are provided to them, they may not actively review or fully recognise their hepatitis status. Studies indicate that approximately 70% of people may have undergone testing multiple times [4, 5]. Hepatitis testing in Japan takes place as part of mandatory health checks [5], before surgeries [4], during pregnancy (HBV) [9], and during blood donation screening (HCV) [9]. In New York City, the cost of duplicate HCV testing was estimated at 1.4 billion dollars without providing additional benefit [10]. In the United States, repeat HBV tests are not recommended unless personal risk profile changes [11]. In Japan, to help reduce the burden of redundant testing, ‘Negative hepatitis virus test result cards’ (NHVC. *Insei*-card in Japanese. *Insei* means negative) were implemented to track the results (Supplementary Figure S1, Additional file 1) [12]. The testing date (year/month/day), the negative result, name, institution, and tester name are written in NHVC by doctors or by the individuals themselves, providing a clear confirmation of HBV/HCV status. Negative test results release the person from the need to undergo other testing; positive test results trigger a referral for further examinations. Aida et al. reported that recognition of negative test results significantly increased from 42% in the first year to 68% in the second year of NHVC distribution [12]. To reduce repeat testing and associated costs to the workplace, efforts are ongoing to promote awareness of the notification and NHVC within companies. However, whether the notification published in 2023 has promoted hepatitis testing and whether NHVC are becoming widely adopted in workplaces remains unknown.

Therefore, we aimed to test three hypotheses: (1) the word awareness rate of the notification and NHVC among stakeholders of a company (exposure) is associated with the rate of providing employees opportunities for hepatitis testing, and (2) correct answers about hepatitis quizzes (outcomes). We also aimed to clarify whether (3) the awareness of employees with HBV/HCV infection (exposure) is associated with word awareness of the notification and NHVC (outcome). In Japan, human resources (HR) personnel are often involved in planning and implementing health checks, including HBV/HCV testing coordination, as well as providing support to employees for balancing work and treatment. While 64.8% of enterprises report using the relationship between in-house HR personnel and other staff to devise work-life balance support measures [13], the company’s policy could be assessed via an online survey aimed at HR personnel only. We believe that, provided HR personnels’ knowledge and awareness are sufficient, this team alone could influence the company’s policy for hepatitis testing. Corporate executives were previously surveyed on in-house policies [14, 15]. Consequently, we decided to include both HR staff and executives as study populations of this survey.

## Methods

### Study design, setting, and period

We conducted an online questionnaire survey in Japan in March 2024. The survey was administered by Cross Marketing, Inc. (Tokyo, Japan). The study has been described previously [16]. This study adhered to the principles of the Declaration of Helsinki. Participants were informed of the study purpose and procedures, and written informed consent was obtained before participation. This study was approved by the Research Ethics Committee of Tokai University, Kanagawa, Japan (protocol number: 23159) on 13 March 2024. This study adhered to the Strengthening the Reporting of Observational Studies in Epidemiology (STROBE) guidelines [17] and checklist (Additional file 2) [18].

### Study population

Cross Marketing utilised participant panels that included connections with executives and HR personnel. Eligible participants were respondents who identified themselves as company executives or HR employees whose job description included labour management or handling medical certificates or both. All questionnaires are shown in the Supplementary Method (Additional file 1).

### Sampling procedure

Participants were screened before the main survey, and those who consented proceeded to the main survey. The sample size was estimated for logistic regression analysis with hepatitis testing as the outcome. We calculated that approximately 1,513 participants were required for this study, assuming a hepatitis testing prevalence of 10% [5], and word awareness prevalence of ≥ 10%, an odds ratio of 2.0, a two-sided significance level of 0.05, and 80% power, using proc power in SAS 9.4 [19]. Our goal was to recruit 1,000 executives and 1,000 HR personnel members. Based on an anticipated response rate of approximately 20%, Cross Marketing sent invitations to approximately 22,000 candidates from 21 to 25 March 2024. The survey was conducted until valid responses of 1,000 executives and 1,000 HR personnel were obtained. Respondents who indicated being ‘executives’ in the screening but reported a non-executive position thereafter were excluded from this study.

### Exposure and outcome assessment

Details of exposure and outcome categories are shown in Table 1. The questionnaires are shown in the Supplementary Method (Additional file 1). Following Trevethan’s framework [20], we categorised ‘word awareness’ and ‘detailed knowledge’ of the notification/NHVC to distinguish the extent to which the policies were known. ‘Word’ awareness of the notification was measured using survey question 9 (SQ9): ‘Did you know that in March 2023, the Ministry of Health, Labour and Welfare issued a notification to labour standards inspection offices instructing them to create opportunities for viral hepatitis testing in the workplace?’ The answers ‘1) I know about it and understand its contents’ and ‘2) I’ve heard of it, but I don’t know the details’ were categorised as ‘having word awareness of notification’; whereas the answer ‘3) I’ve never heard of it’ was categorised as ‘not having word awareness of notification’. Furthermore, answer ‘1) I know about it and understand its contents’ for SQ9 was categorised as ‘having detailed knowledge of notification’ and answers 2) and 3) for SQ9 as ‘not having detailed knowledge of notification’. Word awareness of the NHVC was measured using SQ10: ‘Do you know about the negative hepatitis virus tests result cards? Answers ‘1) I know about it and understand its contents’ and ‘2) I’ve heard of it, but I don’t know the details’ were categorised as ‘having word awareness of NHVC’; whereas ‘3) I’ve never heard of it’ was categorised as ‘not having word awareness of NHVC’. Furthermore, answer ‘1) I know about it and understand its contents’ for SQ10 was categorised as ‘having detailed knowledge of NHVC’ and answers 2) and 3) for SQ10 were categorised as ‘not having detailed knowledge of NHVC’.

**Table 1:** Exposure and outcome category definition

Measurement	Question	Answer	Category
Word awareness of notification	SQ9 Did you know that in March 2023, the Ministry of Health, Labour and Welfare issued a notification to labour standards inspection offices instructing them to create opportunities for viral hepatitis testing in the workplace?	1) I know about it and understand its contents 2) I’ve heard of it, but I don’t know the details	Having word awareness of notification
		3) I’ve never heard of it	Not having word awareness of notification
Detailed knowledge of notification	SQ9 Did you know that in March 2023, the Ministry of Health, Labour and Welfare issued a notification to labour standards inspection offices instructing them to create opportunities for viral hepatitis testing in the workplace?	1) I know about it and understand its contents	Having detailed knowledge of notification
		2) I’ve heard of it, but I don’t know the details 3) I’ve never heard of it	Not having detailed knowledge of notification
Word awareness of NHVC	SQ10 Do you know about the negative hepatitis virus tests result cards?	1) I know about it and understand its contents 2) I’ve heard of it, but I don’t know the details	Having word awareness of NHVC
		3) I’ve never heard of it	Not having word awareness of NHVC
Detailed knowledge of NHVC	SQ10 Do you know about the negative hepatitis virus tests result cards’?	1) I know about it and understand its contents	Having detailed knowledge of NHVC
		2) I’ve heard of it, but I don’t know the details 3) I’ve never heard of it	Not having detailed knowledge of NHVC
Hepatitis testing	SQ3 Does your company conduct hepatitis B and hepatitis C virus testing for employees?	1) All employees are tested simultaneously with their regular health checkups. 2) All employees are tested at a separate time from their regular health checkups. 3) Employees in specific age groups, such as those in their 40s and above, are tested simultaneously with their regular health checkups. 4) Employees in specific age groups (e.g., 40s and above) are tested at a separate time from their regular health checkups. 5) Employees who request it are tested at the same time as their regular health checkup. 6) Employees who request it are tested at a separate time from their regular health checkup. 7) Employees are tested during their initial health checkup upon hiring. 8) Other (each decision).	Conduction of HBV/HCV test
		9) The company does not conduct hepatitis virus testing at any opportunity.	No conduction of HBV/HCV tests
		10) I do not know.	Excluded
Employees with HBV /HCV infection	SQ1 Does your company have any employees who have a history of infection with hepatitis B virus or hepatitis C virus, or who are working while undergoing treatment?	1) Yes	There are employees with HBV /HCV infection
		2) No	No employee with HBV/HCV infection
		3) Unknown	Excluded
Quizzes	SQ7) Now we’ll ask about your knowledge of hepatitis. Read the following statement and select the one you believe is correct. SQ7-1) Among the items currently included in routine health checkups, viral hepatitis can be detected.	3) It can hardly ever be detected in a routine health checkup.	Correct answer
		1) If it’s covered in a routine health checkup, it can be detected nearly 100% of the time. 2) About half (around 50%) of the items covered in a routine health checkup can be detected. 4) It’s unclear whether it can be detected in a routine health checkup.	Not correct
	SQ7-2) Current hepatitis C virus (HCV) infection can be treated.	1) Most (over 95%) can be treated with oral medication.	Correct answer
		2) Most (over 95%) can be treated with injectable medication (interferon). 3) Some people undergo treatment with oral medication. 4) Some people undergo treatment with injectable medication (interferon). 5) It is unclear whether treatment is possible.	Not correct
	SQ7-3) You are going to eat hot pot with someone who has hepatitis C. What is the probability that you will become infected by eating dishes on the same big pot with them?	1) 0%	Correct answer
		2) Around 2% 3) Around 20% 4) Over 80% 5) Don’t know	Not correct

Providing employees with opportunities for hepatitis testing (providing hepatitis testing) was assessed using SQ3: ‘Does your company conduct hepatitis B and hepatitis C virus testing for employees?’ The answers other than ‘9) The company does not conduct hepatitis virus testing at any opportunity’ and ‘10) I don’t know’ were categorised as the existence of opportunities for hepatitis testing, whereas we checked free comments at ‘8) other’ and categorised them respectively (Supplementary Method, Additional file 1). We excluded the respondents who answered ‘10) I don’t know’ in SQ3 from the analysis.

Employees with HBV/HCV infection were assessed using SQ1: ‘Does your company have any employees who have a history of infection with HBV or HCV or who are working while undergoing treatment?’ The answer ‘Yes’ was categorised as ‘there are employees with HBV/HCV infection in the company’.

Quizzes were included in SQ7-1 to SQ7-3, and the number of correct answers was recorded. The questions and correct answers were as follows:

- SQ7: Read the sentences and choose the one you think is correct.
- SQ7-1: Among the items currently included in regular health checks, viral hepatitis can be detected. Correct answer: Regular health checks rarely detect it.
- SQ7-2: Current HCV infection can be treated. Correct answer: Nearly all (over 95%) can be treated with oral medication.
- SQ7-3: You are going to eat hot pot with someone who has hepatitis C. What is the probability that you will become infected by eating dishes on the same big pot with them? Correct answer: 0%.

### Covariates

Executives and HR personnel were categorised by responses to Q4 as ‘2) Executives’ and ‘3) HR personnel’, respectively. Company size was measured using Q9: ‘Please tell us the size of your company (number of employees)’. The answers ‘fewer than 10 people’, ‘10 to 49 people’, ‘50 to 99 people’, ‘100 to 299 people’, ‘300 to 499 people’, ‘500 to 999 people’, and ‘over 1000 people’ were categorised respectively.

### Statistical analysis

First, we examined the characteristics of companies/participants according to word awareness of the notification and NHVC. Subsequently, we examined the association between word awareness of the notification and NHVC as exposures, and providing hepatitis testing as outcome, using logistic regression models, with participants who were not aware of the word of the notification and NHVC as the reference group. We calculated odds ratios (ORs) and 95% confidence intervals (CIs). Model 1 was unadjusted. Model 2 was adjusted for occupation (executives/HR personnel). Model 3 was further adjusted for company size in addition to the adjustments in Model 2.

Moreover, we examined the association between word awareness of the notification and NHVC as exposures, and obtaining one or more correct answers in the quizzes, compared to obtaining no correct answers, as outcomes. The association between word awareness of the notification and NHVC and the respective answers to quizzes was also examined. The association between word awareness of the notification and NHVC and the number of correct answers was also examined using the Cochrane–Mantel–Haenszel tests. We also examined the association between employees with HBV/HCV infection as exposure and word awareness of the notification and NHVC as outcomes. Furthermore, we conducted sensitivity analysis. First, we conducted sensitivity analyses in detailed knowledge of notification or NHVC for SQ9 and SQ10 (Table 1). Furthermore, respondents who answered ‘I don’t know’ in Q9 for screening and SQ1 were included and analysed to assess the robustness of the results.

An alpha level of 0.05 was set, and all p values were two-sided. All statistical analyses were performed using SAS Software version 9.4 (SAS Institute, Cary, NC, USA).

## Results

### Participant characteristics

**Fig. 1:**
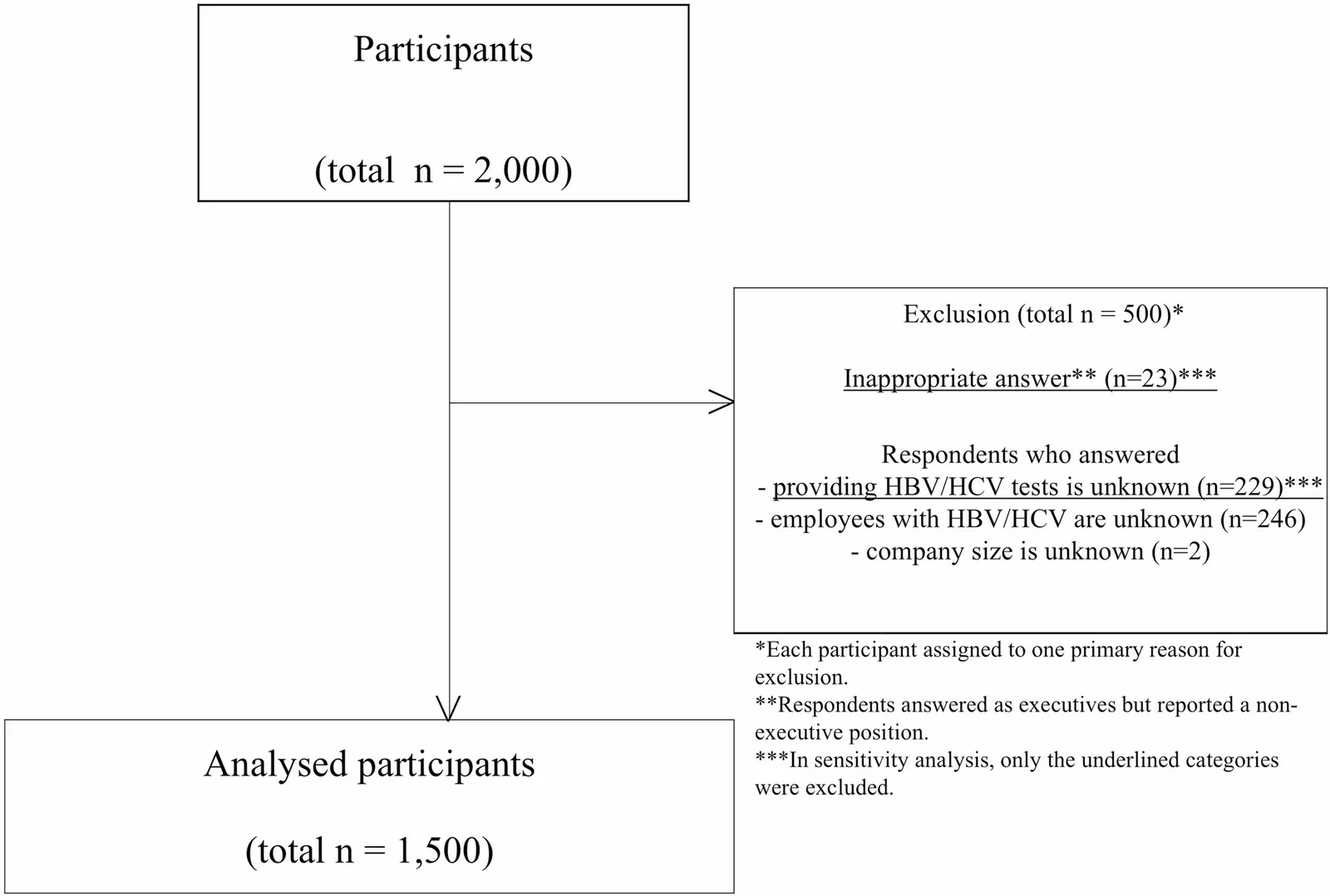
Flow diagram. Flow diagram of study participants. HBV, hepatitis B virus; HCV, hepatitis C virus

A total of 2,000 people (1,000 executives and 1,000 h personnel) responded to the survey, and 1,500 respondents ultimately were included in the analysis. The study flow diagram is shown in Fig. 1, and a detailed flow diagram (including the number of panels) is shown in Supplementary Figure S2 (Additional file 1). Table 2 presents the participants’ background characteristics according to their word awareness of the notification. Participants with word awareness of the notification accounted for 41.8% of the total sample, and HR personnel were more ‘word’ aware than executives (70.3% of the word aware group were HR personnel). Most participants were men, and the mean age [standard deviation] of all participants was 52.5 [11] years. The mean age was similar between the word aware group (52.7 [10.7] years) and unaware group (52.5 [11.1] years). Participants working at large companies were more likely to have word awareness of the notification, with 28.4% of the word aware group employed at companies with 1,000 or more employees. A relatively high proportion of participants worked in construction (word aware group, 7.7%; word unaware group, 10%), manufacturing (15.6%; 15%), and transport and postal services (4.1%; 4.4%), and wholesale and retail trade (8.5%; 11.3%). The proportions of participants covered by in-house health associations was 34.6% in the word aware group and 15.5% in the unaware group, whereas the proportions of participants covered by the Japan Health Insurance Association (Kyokai-kempo) was 34.8% in the word aware group and 55.1% in the unaware group. Supplementary Table S1 (Additional file 1) presents the background characteristics of participants according to their word awareness of NHVC. Participants with word awareness of NHVC accounted for 38% (570 people) of the total sample, and HR personnel were more word aware than executives (71.4% of the word aware group were HR personnel).

**Table 2:** Characteristics of participants and their companies stratified by word awareness of the notification category

	No word awareness of notification	Word awareness of notification
Population, no.	873	627
Male	745 (85.3%)	480 (76.6%)
Age, years	52.5 (± 11.1)	52.7 (± 10.7)
Company size (Number of employees)		
Under 10 persons	363 (41.6%)	72 (11.5%)
10 to 49 persons	156 (17.9%)	67 (10.7%)
50 to 99 persons	75 (8.6%)	68 (10.8%)
100 to 299 persons	112 (12.8%)	111 (17.7%)
300 to 499 persons	36 (4.1%)	68 (10.8%)
500 to 999 persons	47 (5.4%)	63 (10%)
1000 persons or more	84 (9.6%)	178 (28.4%)
Industry		
Agriculture and forestry	3 (0.3%)	4 (0.6%)
Fisheries	1 (0.1%)	2 (0.3%)
Mining and quarrying of stone and gravel	2 (0.2%)	4 (0.6%)
Construction	87 (10%)	48 (7.7%)
Manufacturing	131 (15%)	98 (15.6%)
Electricity, gas, heat supply and water	19 (2.2%)	20 (3.2%)
Information and communications	95 (10.9%)	42 (6.7%)
Transport and postal service	38 (4.4%)	26 (4.1%)
Wholesale and retail trade	99 (11.3%)	53 (8.5%)
Finance and insurance	37 (4.2%)	45 (7.2%)
Real estate and goods rental and leasing	85 (9.7%)	43 (6.9%)
Scientific research, professional and technical services	53 (6.1%)	20 (3.2%)
Accommodations, eating and drinking services	20 (2.3%)	14 (2.2%)
Living-related and personal services and amusement services	21 (2.4%)	20 (3.2%)
Education, learning support	19 (2.2%)	26 (4.1%)
Medical, health care and welfare	42 (4.8%)	67 (10.7%)
Compound services	4 (0.5%)	12 (1.9%)
Service, N.E.C.	117 (13.4%)	83 (13.2%)
Government, except elsewhere classified	0 (0%)	0 (0%)
Founded years		
≤ 3 years	18 (2.1%)	11 (1.8%)
4 to 9 years	82 (9.4%)	64 (10.2%)
10 to 49 years	485 (55.6%)	337 (53.7%)
≥ 50 years	273 (31.3%)	208 (33.2%)
Unknown	15 (1.7%)	7 (1.1%)
Years of working at current firm		
≤ 1 year	22 (2.5%)	14 (2.2%)
1 to 4 years	106 (12.1%)	92 (14.7%)
5 to 9 years	141 (16.2%)	132 (21.1%)
≥ 10 years	604 (69.2%)	389 (62%)
Position		
Executives	609 (69.8%)	190 (30.3%)
Head of a department	58 (6.6%)	136 (21.7%)
Section chief	57 (6.5%)	134 (21.4%)
Managers	52 (6%)	85 (13.6%)
Staff	97 (11.1%)	82 (13.1%)
Health insurance association		
In-house health insurance association	135 (15.5%)	217 (34.6%)
Parent company’s health insurance association	45 (5.2%)	66 (10.5%)
Health insurance association in the industry	105 (12%)	77 (12.3%)
Japan Health Insurance Association (Kyokai-kempo)	481 (55.1%)	218 (34.8%)
Mutual Aid Associations and Mutual Aid Programmes	16 (1.8%)	24 (3.8%)
Seafarers’ Insurance	0 (0%)	3 (0.5%)
National Health Insurance	70 (8%)	16 (2.6%)
Other	1 (0.1%)	1 (0.2%)
Unknown	20 (2.3%)	5 (0.8%)
Occupation		
Human resources	264 (30.2%)	441 (70.3%)
Executive	609 (69.8%)	186 (29.7%)
Employees with HBV or HCV infection		
Yes	55 (6.3%)	258 (41.1%)
No	818 (93.7%)	369 (58.9%)
Hepatitis testing for particular employees		
Yes	244 (27.9%)	522 (83.3%)
No	629 (72.1%)	105 (16.7%)

Percentage may not add up to 100 because of rounding

### Association between word awareness and providing hepatitis testing

Table 3 shows the association between word awareness of the notification and NHVC and providing hepatitis testing. Participants with word aware of the notification had increased odds of providing employees with opportunities for hepatitis tests compared to those without such awareness, after adjustment for occupation and company size (OR = 9.63, 95% CI: 7.34–12.6). Similarly, participants with word awareness of NHVC had higher odds of providing testing for hepatitis than those without such awareness (OR = 10.8, 95% CI: 8.12–14.4).

**Table 3:** Association between word awareness of notification/‘Negative hepatitis virus tests result cards’ and providing hepatitis testing

	Providing employees opportunities for hepatitis testing		Odds ratio (95% confidence interval)		
	No	Yes	Model 1^a)^	Model 2^b)^	Model 3^c)^
The word awareness of notification					
Not aware	629	244	1 (reference)	1 (reference)	1 (reference)
Aware	105	522	12.8 (9.91–16.6)	10.2 (7.84–13.4)	9.63 (7.34–12.6)
Negative hepatitis virus tests result cards					
Not aware	654	276	1 (reference)	1 (reference)	1 (reference)
Aware	80	490	14.5 (11–19.1)	11.6 (8.72–15.3)	10.8 (8.12–14.4)

^a^ Unconditional logistic regression model

^b^ Adjustment for occupation (human resources/executives) in Model 1

^c^Additional adjustment for company size in Model 2

### Association between word awareness and quiz performance

Table 4 shows the association between word awareness of the notification and NHVC and performance on hepatitis-related quizzes. Participants with word awareness of the notification had increased odds of one or more correct answers compared to those without such awareness, after adjustment for occupation and company size (OR = 1.87, 95% CI: 1.49–2.34). Supplementary Table S2 (Additional file 1) shows the association between word awareness of the notification and NHVC and the number of correct quiz answers. The Cochrane–Mantel–Haenszel tests indicated significant associations for both word awareness of the notification and NHVC (*p* < 0.01). Supplementary Table S3 (Additional file 1) shows the association between word awareness of the notification and NHVC and correct answers to quiz questions. For SQ7-2 (treatment of HCV) and SQ7-3 (prejudice toward HCV infection), participants with word awareness of the notification or NHVC were more likely to answer correctly than those without such awareness, after adjustment for occupation and company size (SQ7-2: OR = 2.54, 95% CI: 1.93–3.34 for word awareness of the notification; OR = 2.33, 95% CI: 1.77–3.07 for word awareness of NHVC; SQ7-3: OR = 11.5, 95% CI: 1.16–1.94 in word awareness of notification group; OR = 1.33 95% CI: 1.03–1.73 in word awareness of NHVC group). For SQ7-1 (hepatitis testing in health checkups), participants with word awareness of the NHVC had lower odds of answering correctly than those without such awareness, while the association was not significant for word awareness of the notification.

**Table 4:** Association between word awareness of notification/‘Negative hepatitis virus tests result cards’ and quiz answers

	Correct answers		Odds ratio (95% confidence interval)		
	No	Yes	Model 1^a)^	Model 2^b)^	Model 3^c)^
The word awareness of notification					
Not aware	471	402	1 (reference)	1 (reference)	1 (reference)
Aware	245	382	1.83 (1.48–2.25)	1.87 (1.49–2.34)	1.83 (1.45–2.31)
Negative hepatitis virus tests result cards					
Not aware	481	449	1 (reference)	1 (reference)	1 (reference)
Aware	235	335	1.53 (1.24–1.89)	1.51 (1.2–1.89)	1.45 (1.15–1.83)

^a^Unconditional logistic regression model

^b^Adjustment for occupation (human resources/executives) in Model 1

^c^Additional adjustment for company size in Model 2

### Association between employees with HBV/HCV infection and word awareness

Tables 5 and 6 show the association between the presence of employees with HBV/HCV infection and word awareness of the notification and NHVC. Participants working at companies with employees with HBV/HCV infection were more likely to be aware of the word of notification than those at companies without such employees (OR = 6.42, 95% CI: 4.62–8.93). Similarly, participants at companies with employees with HBV/HCV infection were more likely to be aware of the word of NHVC than those at companies without such employees (OR = 4.9, 95% CI: 3.59–6.68).

**Table 5:** Association between the presence of employees with HBV/HCV infection and word awareness of notification

	The word awareness of notification		Odds ratio (95% confidence interval)		
	Not aware	Aware	Model 1^a)^	Model 2^b)^	Model 3^c)^
HBV/HCV-positive employees					
No	818	369	1 (reference)	1 (reference)	1 (reference)
Yes	55	258	10.4 (7.58–14.3)	7.22 (5.22–9.98)	6.42 (4.62–8.93)

^a^Unconditional logistic regression model

^b^Adjustment for occupation (human resources/executives) in Model 1

^c^Additional adjustment for company size in Model 2

**Table 6:** Association between the presence of employees with HBV/HCV and word awareness of ‘Negative hepatitis virus tests result cards’

	Word of ‘Negative hepatitis virus tests result cards’		Odds ratio (95% confidence interval)		
	Not aware	Aware	Model 1^a)^	Model 2^b)^	Model 3^c)^
HBV/HCV-positive employees					
No	855	332	1 (reference)	1 (reference)	1 (reference)
Yes	75	238	8.17 (6.12–10.9)	5.62 (4.14–7.64)	4.9 (3.59–6.68)

^a^Unconditional logistic regression model

^b^Adjustment for occupation (human resources/executives) in Model 1

^c^Additional adjustment for company size in Model 2

### Sensitivity analysis

Supplementary Table S4 (Additional file 1) shows the association between detailed knowledge of the notification and NHVC and providing testing for hepatitis when detailed knowledge was alternatively categorised (Table 1). The number of participants with detailed knowledge of the notifications and NHVC was 190 (12.6%) and 182 (12.1%), respectively. Participants in the detailed knowledgeable group were more likely to provide opportunities for hepatitis testing compared to those in the non-detailed knowledgeable group, after adjustment for occupation and company size (detailed knowledge of the notification: OR = 26.6, 95% CI: 16.6–60.9; detailed knowledge of NHVC: OR = 24.4, 95% CI: 10.7–56.1).

Supplementary Table S5 (Additional file 1) presents the characteristics of participants and their companies, excluding individuals with inappropriate answers and missing information about providing employees with opportunities for hepatitis testing. In this group, 1,045 participants lacked word awareness of the notification, while 703 had word awareness of the notification. Supplementary Table S6 (Additional file 1) shows that participants with word awareness of the notification or NHVC were more likely to provide employees with opportunities for hepatitis testing than those without word awareness, after adjustment for occupation and company size (word awareness of the notification: OR = 9.4, 95% CI: 7.4–12; word awareness of NHVC: OR = 10.7, 95% CI: 8.28–13.9).

## Discussion

In this study, we developed an online questionnaire for company executives and HR personnel regarding providing employees with opportunities for hepatitis testing and word awareness of the notification and NHVC. HR personnel were more aware about the word of the notification and NHVC than executives. Our study results showed a significant association between word awareness of the notification and NHVC and providing employees with opportunities for hepatitis testing. Additionally, participants who had awareness of the word of notification or NHVC were significantly more likely to answer hepatitis-related quizzes correctly. Participants working in companies with employees with HBV/HCV infection had more awareness of the word of the notification or NHVC. The results of the sensitivity analysis were consistent. Participants classified as knowledgeable about details of the notification or NHVC were more likely to provide hepatitis tests.

This study demonstrated that the participants at companies providing hepatitis testing opportunities were more aware of the notification and NHVC, including their details [5]. This study is first to examine the impact of government notification on public awareness, typically for HR staffs and executives, which could affect OHS policies. Tompa et al. categorised nine clusters of studies on OHS policies, and this study would be categorised as an ‘awareness campaign’. The authors concluded that interventions such as inspections [21], training [22], and distribution of booklets [23] had moderate evidence for improving awareness and compliance regarding OHS, whereas they showed limited evidence for reducing injuries [7]. Additionally, Andersen conducted a systematic review of these interventions, including studies on written materials and broadcasting, showing moderately strong evidence for reductions in injuries and improved compliance by OHS [24]. Therefore, stakeholders in companies that have implemented hepatitis testing opportunities may be more information-sensitive. Our results provide insight into the importance of raising awareness among companies’ stakeholders for providing employees with opportunities to undergo hepatitis screening. Our previous study showed that HBV/HCV incidence was associated with occupation and industry categories [25]. Implementing hepatitis testing and raising awareness could be combined with internal drives by HR personnel and executives marketing’ the relevant policies [16]. Further intervention studies are needed to clarify the effectiveness of notifications and NHVC.

In addition, providing opportunities for hepatitis testing might increase notification and NHVC awareness and vice versa. However, hepatitis is a blood-borne disease and knowing one’s disease status is not equivalent to undergoing treatment. These findings suggest the importance of screening; however, the pathway from workplace-based screening to clinical diagnosis and treatment is unclear. Some municipalities follow residents’ appointment and treatment status [4, 26]. Workplaces should implement active follow-up programs to ensure that testing effectively translates into proper treatment and prevention.

The quizzes assessed prejudice toward HCV infection (SQ7-3) and revealed that participants with awareness of the notification or NHVC were more likely to answer correctly. We previously conducted similar quizzes regarding prejudice for hepatitis among the general population (*n* = 500), with a correct answer rate of 71.6% [27]. The previous question was easier than the question in the current survey because it required only yes or no answers, whereas the present survey included four response options (Supplementary Table S7, Additional file) [27]. We included HR personnel whose main responsibilities involve labour management or reviewing medical certifications. Given that their roles required medical literacy, including safety and hygiene, they could collect information about providing workers with opportunities for hepatitis testing. Meanwhile, Eguchi et al. showed that knowledge about HBV/HCV may decrease individuals’ negative attitudes toward colleagues with HBV/HCV infection [28]. More recently, Yatsuhashi et al. built a homepage providing information on prejudice against people with HBV/HCV infection [29]. In this study, respondents who answered they knew other employees with HBV/HCV were more likely to be aware of the word of notification and NHVC (Tables 5 and 6). Stakeholders in companies having employees with HBV/HCV infection may collect information regarding hepatitis and prejudice. In addition, the group with awareness of the notification/NHVC was more likely to correctly answer question SQ7-3 about the misconception of transmission (Supplementary Table S3). The notification/NHVC could help improve the public knowledge base and reduce general prejudice associated with HBV/HCV infection by promoting accurate information and testing. Concurrently, worker privacy should be protected, and employees should have the right to refuse sharing their test results with employers [4]; barriers to testing uptake should be further examined. A 2011 study showed that approximately 35% workers were anxious about becoming infected from colleagues with hepatitis [30]. Harris et al. showed in quantitative research that patients with HBV or HCV infection chose non-disclosure because of stigma [31]. To decrease workplace stigma and help increase the currently low screening coverage, we recommend that workers be encouraged to carry their NHVC alongside their medication record handbooks [12, 32]. Normalising this practice may lower psychological barriers to testing, while encouraging workers with positive results to seek treatment.

This study has two strengths. First, this is the first survey to demonstrate the association between the notification and NHVC and providing hepatitis testing. Second, participants were recruited through participant panels, thereby ensuring the accuracy of their occupational information.

This study also has some limitations. First, because the survey was conducted online, selection bias in participants’ backgrounds may have occurred. Although it is difficult to generalise the findings to all Japanese companies because of the sampling bias, we conducted sensitivity analyses to confirm the association between awareness of the notification and NHVC and providing hepatitis testing. Second, this was a cross-sectional survey; hence, we could not determine causal relationships or changes in awareness or providing hepatitis testing rates over time. Future studies should verify these findings using designs and analyses that control for confounding factors. Finally, other unmeasured confounders may exist. Further studies, including follow-up studies, are needed to clarify the causal relationship between awareness of the notification or NHVC and providing hepatitis testing.

## Conclusions

This study showed that executives and HR personnel with awareness of the word of the notification or NHVC provided employees more opportunities for hepatitis testing. These findings may support the promotion of the notification and NHVC among executives and personnel, helping to expand hepatitis screening opportunities in the workplace. Using marketing tools or other strategies, further studies evaluating the awareness and implementation of the notification/NHVC, as well as barriers to uptake, are required in workplaces.

## Supplementary Information

Additional file 1: Supplementary Figure S1: Negative hepatitis virus tests result cards (NHVC). Supplementary. Figure S2: Detailed flow diagram. Supplementary methods: The questionnaire. Supplementary Table S1: Background characteristics based on word awareness of ‘Negative hepatitis virus tests result cards’. Supplementary Table S2: Association between correct quiz answers and word awareness of the notification or ‘Negative hepatitis virus tests result cards’. Supplementary Table S3: Association between correct quiz answers and word awareness of the notification or ‘Negative hepatitis virus tests result cards’. Supplementary Table S4: Association between detailed knowledge of the notification or ‘Negative hepatitis virus tests result cards’ and hepatitis testing in sensitivity analysis (Analysing the detailed knowledge of the notification or ‘Negative hepatitis virus tests result cards’ categories in Table 1). Supplementary Table S5: Characteristics of participants and their companies in sensitivity analysis (population only excluded participants with inappropriate answers and unknown hepatitis test status) stratified by word awareness of the notification. Supplementary Table S6: Association between word awareness of the notification / ‘Negative hepatitis virus tests result cards’ and testing for hepatitis in sensitivity analysis (population only excluded participants with inappropriate answers and unknown hepatitis test status). Supplementary Table S7: Comparison of hepatitis quizzes.

Additional file 2: The STROBE reporting checklist.

## Data Availability

The datasets analysed during this study are not publicly available due to licencing restrictions but are available from the corresponding author on reasonable request.

## Abbreviations

CI: Confidence interval
HBV: Hepatitis B virus
HCV: Hepatitis C virus
HR: Human resources
NHVC: Negative hepatitis virus tests result cards
OHS: Occupational health and safety
