# Supplementary material for: Association between awareness of government notification and ‘negative hepatitis virus test result card’ and implementation of hepatitis B and C screening in Japanese workplaces: a cross-sectional study
Source: BMC Public Health. 2026 Aug 7;26:3188. doi: 10.1186/s12889-026-28932-7 (PMC13729562; doi:10.1186/s12889-026-28932-7)
Supplement: Supplementary file 1 [file 12889_2026_28932_MOESM1_ESM.docx]

Supplementary file:

Shoko Nakazawa, Kosuke Sakai, Kota Fukai, Yuko Furuya, Kei Sano, Masayuki Tatemichi, and Masaaki Korenaga

**Association between awareness of government notification and ‘Negative hepatitis virus test result card’ and implementation of hepatitis B and C screening in Japanese workplaces: A cross-sectional study**

Contents

[Supplementary Figure S1 ‘Negative hepatitis virus tests result cards’ (NHVC)	2](#_Toc227662727)

[Supplementary Figure S2 Detailed flow diagram	3](#_Toc227662728)

[Supplementary methods: The questionnaire	4](#_Toc227662729)

[Supplementary Table S1 Characteristics of participants and their companies stratified by word awareness of ‘Negative hepatitis virus tests result cards’	24](#_Toc227662730)

[Supplementary Table S2 Association between correct answers in quizzes and word awareness of the notification or ‘Negative hepatitis virus tests result cards’	29](#_Toc227662731)

[Supplementary Table S3 Association between correct quiz answers and word awareness of the notification or ‘Negative hepatitis virus tests result cards’	30](#_Toc227662732)

[Supplementary Table S4 Association between content awareness of the notification or ‘Negative hepatitis virus tests result cards’ and testing for hepatitis in sensitivity analysis (analysing the content awareness of the notification or ‘Negative hepatitis virus tests result cards’ categories in Table 1).	33](#_Toc227662733)

[Supplementary Table S5 Characteristics of participants and their companies in sensitivity analysis (population only excluded participants with inappropriate answers and unknown hepatitis test status) stratified by word awareness of the notification	35](#_Toc227662734)

[Supplementary Table S6 Association between word awareness of the notification or ‘Negative hepatitis virus tests result cards’ and testing for hepatitis in sensitivity analysis (population only excluded participants with inappropriate answers and unknown hepatitis test status)	39](#_Toc227662735)

[Supplementary Table S7 Comparison of hepatitis quizzes	41](#_Toc227662736)

# Supplementary Figure S1 Negative hepatitis virus tests result cards (NHVC) ^a)^

Description:

The hepatitis virus test results are negative.

Confirmed date (year/month/day)

HBs antigen (+ -) HCV antibody (+ -)

Name

Institution

Verifier’s name

Please present this card when receiving medical examinations or tests at hospitals or dental clinics.

a) Aida M, Ikegami T, Korenaga M, Ministry of Health, Labor and Welfare, “Research Group for Improving the Hepatitis Virus Test Acceptance Rate and the Receiving Medical Care Acceptance Rate of Positive Patients Using New Methods.” Methods of communicating negative results of hepatitis virus tests. Kanzo. 2023;64:259-69.

Available from: https://kanzochan.jp/goods/dl/hepatitis_negative.pdf

# Supplementary Figure S2 Detailed flow diagram

People in the panel

Executives: 20,579

Human resources: 30,624

Exclusion (Total n=500*)

Inappropriate answer** (n=23)***

Respondents who answered

- testing of HBV /HCV is unknown (n=229)***

- employees with HBV /HCV are unknown (n=246)

- company size is unknown (n=2)

*Each participant assigned to one primary reason for exclusion.

**Respondents answered as executives but reported a non-executive position.

***In sensitivity analysis, only the underlined categories were excluded.

Invitation sent to 22,818 people with the following characteristics:

Residence: nationwide in Japan

Sex: Male or Female

Age: 20 to 69 years

Other information: based on their position/job type information

People who answered screening questions: 4,196

Participants: 2,000

Analysed participants: 1,500

People who are not matched to eligible criteria: 1,659

Incomplete response or dropout; 97

# Supplementary methods: The questionnaire. **Bold and underline** show the relevant questions to this study.

**Screening section**

**Q1_1) Please tell me your age.** A. (number) years

**Q2) Please tell me your sex.**

1. Male
2. Female
3. I don't want to answer.

Q3) Please tell me where you live. A. Prefecture name

**Q4) Please tell me your current occupation.**

1) Government staff
2) Executive
3) Employee (human resources or general affairs)
4) Employee (sales)
5) Employee (engineering)
6) Employee (other fields)
7) Self-employed
8) Freelance
9) Part-timer
10) Homemaker
11) Student
12) Unemployed

**Q5) Please answer this question only when you chose 3) Employee (human resources or general affairs) in Q4. What is your main job description?**

1)	Labour management

2)	Recruitment

3)	Education and training

4)	General affairs

5)	Other

**Q6) Please answer this question only when you chose 3) Employee (human resources or general affairs) in Q4. Are you able to see medical certification of employees?**

1)	Yes

2)	No

**Q7) Please answer this question only when you chose 3) Employee (human resources or general affairs) in Q4. Do you handle labour management?**

1)	Yes

2)	No

Q8) What type of industry does your company operate in? (Japan Standard Industrial Classification, major classification).

1A Agriculture and forestry

2B Fishing

3C Mining, quarrying, and gravel extraction

4D Construction

5E Manufacturing

6F Electricity, gas, heat supply, and water supply

7G Information and communication industry

8H Transportation and postal industry

9I Wholesale and retail trade

10J Financial industry and insurance industry

11K Real estate and goods rental industry

12L Academic research, professional and technical services

13M Accommodation, food services

14N Lifestyle-related services, entertainment

15O Education and learning support

16P Medical care, welfare

17Q Complex service businesses

18R Services, not elsewhere classified

19S Public services, except elsewhere classified

20T Industries not classified (including unemployed, housewives, and students)

21U Unknown

**Q9) What is the size of your company?**

1) 1 to 9 people
2) 10 to 49 people
3) 50 to 99 people
4) 100 to 299 people
5) 300 to 499 people
6) 500 to 999 people
7) Over 1000 people
8) Unknown

**Main section**

**SQ1) Does your company have any employees who have a history of infection with hepatitis B virus (HBV) or hepatitis C virus (HCV), or who are working while undergoing treatment?**

1)	Yes

2)	No

3)	Unknown

SQ2) Please answer this question only when you chose 1) Yes in SQ1.

When did you know the presence of employees with HBV/HCV? (choose multiple)

1) When I looked at the results of his / her hepatitis test in annual health check-ups or Ningen Dock.
2) When I looked at the questionnaire of his / her annual check-ups or Ningen Dock.
3) I heard directly from the employee (I did not receive the medical certificate).
4) I heard rumours of the employee (I did not receive the medical certificate).
5) The medical certificate was submitted when he / she received treatment for hepatitis.
6) Other (free form)
7) I don't remember.

**SQ3) Does your company conduct hepatitis B and hepatitis C virus testing for employees? (choose multiple)**

1) All employees are tested simultaneously with their regular health checkups.

2) All employees are tested at a separate time from their regular health checkups.

3) Employees in specific age groups, such as those in their 40s and above, are tested simultaneously with their regular health checkups.

4) Employees in specific age groups (e.g. 40s and above) are tested at a separate time from their regular health checkups.

5) Employees who request it are tested at the same time as their regular health checkup.

6) Employees who request it are tested at a separate time from their regular health checkup.

7) Employees are tested during their initial health checkup upon hiring.

8) Other (free form)

9) The company does not conduct hepatitis virus testing at any opportunity it provides.

10) Unknown

SQ4) Please answer this question only when you choose 1) to 8) in SQ3. Who covers the cost of testing for hepatitis B virus and hepatitis C virus mentioned in SQ3?

1) The company covers the full cost of the examination, with no out-of-pocket expense for the employee.
2) The health insurance association covers the full cost of the examination, with no out-of-pocket expense for the employee.
3) The company and health insurance association share the cost of the examination, with no out-of-pocket expense for the employee.
4) The company covers part of the examination cost, with the remainder being the employee's out-of-pocket expense.
5) The health insurance association covers part of the examination cost, with the remainder being the employee's out-of-pocket expense.
6) The employee pays the full cost of the examination.
7) Other (free form)
8) Unknown

SQ5) Who at your company keeps track of the results of health checkups and comprehensive medical examinations? (choose multiple)

1) All results from health checkups and comprehensive medical examinations are managed by the HR personnel (health checkup coordinator).
2) All results from health checkups and comprehensive medical examinations are managed by the company physician.
3) All results from health checkups and comprehensive medical examinations are managed by the occupational health nursing staff (nurses/public health nurses).
4) All results from health checkups and comprehensive medical examinations are managed by the safety manager.
5) Only the statutory items under the Industrial Safety and Health Act from health checkups and comprehensive medical checkups are known to the personnel manager (health checkup coordinator).
6) Only the statutory items under the Industrial Safety and Health Act from health checkups and comprehensive medical checkups are known to the company doctor.
7) Only the statutory items under the Industrial Safety and Health Act from health checkups and comprehensive medical checkups are known to the occupational health nursing staff (nurses/public health nurses).
8) Only the statutory items under the Industrial Safety and Health Regulations from health checkups and comprehensive medical checkups are managed by the health and safety manager.
9) The results of health checkups and comprehensive medical checkups are only stored by the company; no one has access to the results.
10) Other (free form)
11) Unknown

SQ6) Does your company receive support from occupational health staff (occupational physicians and occupational health nurses/public health nurses) regarding the planning and implementation of hepatitis testing? Please select one option for each.

SQ6-1) about a dedicated full-time occupational physician

1) You can get proactive involvement

2) You can get support if you ask

3) Limited time and resources, so you can't ask

4) Hardly any support available

5) Not sure

6) Not available / No contract

SQ6-2) about a part-time occupational physician

1) You can get proactive involvement

2) You can get support if you ask

3) Limited time and resources, so you can't ask

4) Hardly any support available

5) Not sure

6) Not available / No contract

SQ6-3) about an occupational physician at the parent company

1) You can get proactive involvement

2) You can get support if you ask

3) Limited time and resources, so you can't ask

4) Hardly any support available

5) Not sure

6) Not available / No contract

SQ6-4) about occupational health nurses (public health nurses/registered nurses)

1) You can get proactive involvement

2) You can get support if you ask

3) Limited time and resources, so you can't ask

4) Hardly any support available

5) Not sure

6) Not available / No contract

SQ6-5) about contract-based occupational health nurses (public health nurses/registered nurses) at the company

1) You can get proactive involvement

2) You can get support if you ask

3) Limited time and resources, so you can't ask

4) Hardly any support available

5) Not sure

6) Not available / No contract

SQ6-6) Occupational health nurses (public health nurses/registered nurses) at the parent company

1) You can get proactive involvement

2) You can get support if you ask

3) Limited time and resources, so you can't ask

4) Hardly any support available

5) Not sure

6) Not available / No contract

SQ6-7) Occupational physicians and occupational health nurses/public health nurses at health checkup examination

1) You can get proactive involvement

2) You can get support if you ask

3) Limited time and resources, so you can't ask

4) Hardly any support available

5) Not sure

6) Not available / No contract

**SQ7) Now we'll ask about your knowledge of hepatitis. Read the following statement and select the one you believe is correct.**

**SQ7-1) Among the items currently included in routine health checkups, viral hepatitis can be detected.**

1) If it's included in a routine health checkup, it can be detected nearly 100% of the time.

2) About half (around 50%) of the items covered in a routine health checkup can be detected.

3) It can hardly ever be detected in a routine health checkup.

4) It's unclear whether it can be detected in a routine health checkup.

**SQ7-2) Current hepatitis C virus (HCV) infection can be treated.**

1) Most (over 95%) can be treated with oral medication.
2) Most (over 95%) can be treated with injectable medication (interferon).
3) Some people undergo treatment with oral medication.
4) Some people undergo treatment with injectable medication (interferon).
5) It is unclear whether treatment is possible.

**SQ7-3) You are going to eat hot pot with someone who has hepatitis C. What is the probability that you will become infected by eating dishes on the same big pot with them?**

1) 0%
2) Around 2%
3) Around 20%
4) Over 80%
5) Don't know

SQ8) How often do you think hepatitis virus testing should be performed?

1) Annual health examination are required.
2) A checkup is required once every ten years.
3) You only need to get tested once in your lifetime.
4) Don't know

**SQ9) Did you know that in March 2023, the Ministry of Health, Labour and Welfare issued a notification to labour standards inspection offices instructing them to create opportunities for viral hepatitis testing in the workplace?**

1) I know about it and understand its contents.
2) I've heard of it, but I don't know the details.
3) I've never heard of it.

**SQ10) Do you know about negative hepatitis virus tests result cards?**

1) I know about it and understand its contents.
2) I've heard of it, but I don't know the details.
3) I've never heard of it.

SQ11-1) Are you aware of the existence of the following internet sites?

The Ministry of Health, Labour and Welfare homepage's ‘Balancing Treatment and Work’ https://www.mhlw.go.jp/stf/seisakunitsuite/bunya/0000115267.html

1) I know about it and I’ve seen it.
2) I've heard of it, but I’ve never seen it.
3) I've never heard of it.

SQ11-2) Are you aware of the existence of the following internet sites?

Homepage ‘Hepatitis Information Center, The Research Center for Hepatitis and Immunology, National Institute of Global Health and Medicine, Japan Institute for Health Security (JIHS)’

(In the questionnaire, the URL was https://www.kanen.ncgm.go.jp/index.html but the homepage has been moved to https://www.kanen.jihs.go.jp/)

1) I know about it and I’ve seen it.
2) I've heard of it, but I’ve never seen it.
3) I've never heard of it.

SQ11-3) Are you aware of the existence of the following internet sites?

Homepage ‘Aware, Hepatitis’ https://www.kanen.org/

1) I know about it and I’ve seen it.
2) I've heard of it, but I’ve never seen it.
3) I've never heard of it.

SQ11-4) Are you aware of the existence of the following internet sites?

Homepage ‘Hepatitis Medical Navigation System (Kan-Navi)’

(In the questionnaire, the URL was https://kan-navi.jihs.go.jp/index-s.html but the homepage has been moved to https://kan-navi.jihs.go.jp/)

1) I know about it and I’ve seen it.
2) I've heard of it, but I’ve never seen it.
3) I've never heard of it.

SQ11-5) Are you aware of the existence of the following internet sites?

‘Hepatitis Testing and Treatment Guide for Workers: Supporting the Balance Between Work and Treatment’ https://kanen5.med.u-tokai.ac.jp/library.html

1) I know about it and I’ve seen it.
2) I've heard of it, but I’ve never seen it.
3) I've never heard of it.

SQ11-6) Are you aware of the existence of the following internet sites?

‘Hepatitis.net’ https://www.kanen-net.info/

(the homepage was closed accessed 09 Jan 2026)

1) I know about it and I’ve seen it.
2) I've heard of it, but I’ve never seen it.
3) I've never heard of it.

SQ12) If you were planning to conduct hepatitis virus testing at your company, what information would help you plan the test?

1) List of medical facilities and health screening centres where hepatitis virus testing is available; Testing agency

2) Frequency and timing of hepatitis virus testing; Testing information

3) List of medical institutions where you can be examined when you are found to be infected with hepatitis virus; Medical facilities

4) How to treat an employee who is found to be positive for the hepatitis virus test; Communication

5) The impact on work when treating for viral hepatitis; Job impact

6) How to store the results of medical examinations, including hepatitis tests; Result storage

7) How to protect personal information such as hepatitis test results; Privacy

8) How to deal with prejudice against the hepatitis virus; Prejudice Management

9) How to create a support plan for balancing hepatitis treatment and work; Support plan, 10) How to create an in-house system for balancing hepatitis treatment and work; Treatment and Work Balance System

11) Other (free form)

12) Nothing in particular

13) Unknown

SQ13) This question is not limited to hepatitis information, but which of the following sources of information do you use to obtain information for your business?

1) The Nikkei and Nikkei Digital Edition
2) Various newspapers other than The Nikkei
3) Magazines targeting HR and general affairs personnel
4) Specialised journals related to HR and social insurance
5) Magazines other than those targeting HR or specialised journals
6) HR portal sites (e.g. ‘Japan's HR Department’)
7) Email newsletters from HR system companies (e.g. attendance management systems)
8) Opinions of senior colleagues and supervisors in HR and general affairs departments
9) Information from HR and general affairs departments at other companies
10) Opinions of legal advisors and social insurance and labour consultants
11) Opinions of occupational physicians and occupational health nurses (public health nurses/registered nurses)
12) Opinions of health insurance association representatives
13) Websites of public institutions such as the Ministry of Health, Labour and Welfare
14) Search results from search engines (Google, Yahoo!, etc.)
15) Online advertisements
16) Offline advertising (brochures, catalogues, flyers, etc.)
17) SNS posts/photos (from official company accounts)
18) SNS posts/photos (from acquaintances/friends connected via SNS, e.g. via follow or friend requests)
19) SNS posts/photos (from other individuals not connected via SNS)
20) Other (free form)
21) No specific information sources

SQ14) This question is not limited to hepatitis information, but which of

the following sources of information do you use to obtain information for your business?

SQ14-1) NIKKEI

1) Regularly view
2) Occasionally view
3) View when needed
4) Never view

SQ14-2) Web Rodo Seisaku Jihou

1) Regularly view
2) Occasionally view
3) View when needed
4) Never view

SQ14-3) Jinji-bu of Japan

1) Regularly view
2) Occasionally view
3) View when needed
4) Never view

SQ14-4) HR Pro

1) Regularly view
2) Occasionally view
3) View when needed
4) Never view

SQ14-5) Recruit Works

1) Regularly view
2) Occasionally view
3) View when needed
4) Never view

SQ14-6) HR Mikata

1) Regularly view
2) Occasionally view
3) View when needed
4) Never view

SQ14-7) BizHint

1) Regularly view
2) Occasionally view
3) View when needed
4) Never view

SQ14-8) YouTube

1) Regularly view
2) Occasionally view
3) View when needed
4) Never view

SQ14-9) SNS; LINE

1) Regularly view
2) Occasionally view
3) View when needed
4) Never view

SQ14-10) SNS; Facebook

1) Regularly view
2) Occasionally view
3) View when needed
4) Never view

SQ14-11) SNS; Instagram

1) Regularly view
2) Occasionally view
3) View when needed
4) Never view

SQ14-12) SNS; X (Twitter)

1) Regularly view
2) Occasionally view
3) View when needed
4) Never view

SQ14-13) SNS and Other (free form)

1) Regularly view
2) Occasionally view
3) View when needed
4) Never view

**SQ15) How many years has your company been in business?**

1) Less than 3 years

2) 3 to 9 years

3) 10 to 49 years

4) More than 50 years

5) Don't know

**SQ16) How long have you been with your current company?**

1) Less than 1 year
2) 1 to 4 years
3) 5 to 9 years
4) 10 years or more

**SQ17) What is your job title?**

1) Director/Officer

2) Department manager

3) Section chief

4) Section manager/Chief

5) General employee/person in charge

**SQ18) Which is your company's health insurance?**

1) Own health insurance association

2) Parent company's health insurance association

3) Health insurance associations for each industry (Kanto IT Software Health Insurance Association, Publication Health Insurance Association, etc.)

4) National Health Insurance Association (Kyokai kenpo)

5) Mutual aid association/ mutual aid system

6) Seafarers' insurance

7) National Health Insurance

8) Other (free form)

9) I don’t know.

# Supplementary Table S1 Background characteristics based on word awareness of ‘Negative hepatitis virus tests result cards’

|  |  | Not having awareness of the word of ‘Negative hepatitis virus tests result cards’ | | Having awareness of the word of ‘Negative hepatitis virus tests result cards’ | |
| --- | --- | --- | --- | --- | --- |
| Population, no. |  | 930 | | 570 | |
| Male |  | 789 (84.8%) | | 436 (76.5%) | |
| Age, years |  | 54.6 (±10) | | 49.1 (±11.7) | |
| Company size (Number of employees) |  |  | |  | |
| Under 10 persons |  | 378 (40.6%) | | 57 (10%) | |
| 10 to 49 persons |  | 160 (17.2%) | | 63 (11.1%) | |
| 50 to 99 persons |  | 83 (8.9%) | | 60 (10.5%) | |
| 100 to 299 persons |  | 122 (13.1%) | | 101 (17.7%) | |
| 300 to 499 persons |  | 43 (4.6%) | | 61 (10.7%) | |
| 500 to 999 persons |  | 51 (5.5%) | | 59 (10.4%) | |
| 1000 persons or more |  | 93 (10%) | | 169 (29.6%) | |
| Industry |  |  | |  | |
| Agriculture and forestry |  | 4 (0.4%) | | 3 (0.5%) | |
| Fisheries |  | 1 (0.1%) | | 2 (0.4%) | |
| Mining and quarrying of stone and gravel |  | 2 (0.2%) | | 4 (0.7%) | |
| Construction |  | 90 (9.7%) | | 45 (7.9%) | |
| Manufacturing |  | 137 (14.7%) | | 92 (16.1%) | |
| Electricity, gas, heat supply and water |  | 19 (2%) | | 20 (3.5%) | |
| Information and communications |  | 96 (10.3%) | | 41 (7.2%) | |
| Transport and postal service |  | 42 (4.5%) | | 22 (3.9%) | |
| Wholesale and retail trade |  | 105 (11.3%) | | 47 (8.2%) | |
| Finance and insurance |  | 41 (4.4%) | | 41 (7.2%) | |
| Real estate and goods rental and leasing |  | 89 (9.6%) | | 39 (6.8%) | |
| Scientific research, professional and technical services | 53 (5.7%) | | 20 (3.5%) | |  |
| Accommodations, eating and drinking services | 19 (2%) | | 15 (2.6%) | |  |
| Living-related and personal services and amusement services | 24 (2.6%) | | 17 (3%) | |  |
| Education, learning support |  | 22 (2.4%) | | 23 (4%) | |
| Medical, health care and welfare |  | 50 (5.4%) | | 59 (10.4%) | |
| Compound services |  | 4 (0.4%) | | 12 (2.1%) | |
| Service, N.E.C. |  | 132 (14.2%) | | 68 (11.9%) | |
| Government, except elsewhere classified |  | 0 (%) | | 0 (%) | |
| Founded years |  |  | |  | |
| 3 years or less |  | 20 (2.2%) | | 9 (1.6%) | |
| 4 to 9 years |  | 82 (8.8%) | | 64 (11.2%) | |
| 10 to 49 years |  | 530 (57%) | | 292 (51.2%) | |
| 50 years or more |  | 281 (30.2%) | | 200 (35.1%) | |
| Unknown |  | 17 (1.8%) | | 5 (0.9%) | |
| Years of working at current firm |  |  | |  | |
| 1 year or less |  | 28 (3%) | | 8 (1.4%) | |
| 1 to 4 years |  | 115 (12.4%) | | 83 (14.6%) | |
| 5 to 9 years |  | 142 (15.3%) | | 131 (23%) | |
| 10 years or more |  | 645 (69.4%) | | 348 (61.1%) | |
| Position |  |  | |  | |
| Executives |  | 633 (68.1%) | | 166 (29.1%) | |
| Head of department |  | 65 (7%) | | 129 (22.6%) | |
| Section chief |  | 65 (7%) | | 126 (22.1%) | |
| Managers |  | 53 (5.7%) | | 84 (14.7%) | |
| Staff |  | 114 (12.3%) | | 65 (11.4%) | |
| Health insurance association |  |  | |  | |
| In-house health insurance association |  | 150 (16.1%) | | 202 (35.4%) | |
| Parent company's health insurance association | 56 (6%) | | 55 (9.6%) | |  |
| Health insurance association in the industry |  | 111 (11.9%) | | 71 (12.5%) | |
| Japan Health Insurance Association (Kyokai-Kempo) |  | 509 (54.7%) | | 190 (33.3%) | |
| Mutual Aid Associations and Mutual Aid Programmes | 14 (1.5%) | | 26 (4.6%) | |  |
| Seafarers’ Insurance |  | . (.%) | | 3 (0.5%) | |
| National Health Insurance |  | 69 (7.4%) | | 17 (3%) | |
| Other |  | 2 (0.2%) | | . (.%) | |
| Unknown |  | 19 (2%) | | 6 (1.1%) | |
| Occupation |  |  | |  | |
| Human resources |  | 298 (32%) | | 407 (71.4%) | |
| Executive |  | 632 (68%) | | 163 (28.6%) | |
| Employees with HBV or HCV infection |  |  | |  | |
| Yes |  | 75 (8.1%) | | 238 (41.8%) | |
| No |  | 855 (91.9%) | | 332 (58.2%) | |
| Hepatitis testing for particular employees |  |  | |  | |
| Yes |  | 276 (29.7%) | | 490 (86%) | |
| No |  | 654 (70.3%) | | 80 (14%) | |

Percentage may not add up to 100 because of rounding.

# Supplementary Table S2 Association between correct quiz answers and word awareness of the notification or ‘Negative hepatitis virus tests result cards’

|  | Number of correct answers | | | | p-value ^a)^ |
| --- | --- | --- | --- | --- | --- |
|  | 0 | 1 | 2 | 3 |  |
| The word awareness of notification |  |  |  |  |  |
| Not aware | 471 | 269 | 118 | 15 |  |
| Aware | 245 | 249 | 123 | 10 | <0.001 |
| The word awareness of ‘Negative hepatitis virus tests result cards’ |  |  |  |  |  |
| Not aware | 481 | 302 | 131 | 16 |  |
| Aware | 235 | 216 | 110 | 9 | <0.001 |

a) Cochrane–Mantel–Haenszel statistics.

# Supplementary Table S3 Association between correct quiz answers and word awareness of the notification or ‘Negative hepatitis virus tests result cards’

|  | A correct answer for SQ7-1  (Among the items currently included in regular health checkups, viral hepatitis can be detected.) | | Odds ratio (95% CI) ^a)^ |
| --- | --- | --- | --- |
| The word awareness of notification | No | Yes |  |
| Not aware | 703 | 170 | 1 (reference) |
| Aware | 519 | 108 | 0.79 (0.58–1.07) |
| The word awareness of ‘Negative hepatitis virus tests result cards’ |  |  |  |
| Not aware | 740 | 190 | 1 (reference) |
| Aware | 482 | 88 | 0.63 (0.46–0.86) |
|  | A correct answer for SQ7-2  (Current HCV infection can be treated.) | | Odds ratio (95% CI) ^a)^ |
| The word awareness of notification | No | Yes |  |
| Not aware | 731 | 142 | 1 (reference) |
| Aware | 419 | 208 | 2.54 (1.93–3.34) |
| The word awareness of ‘Negative hepatitis virus tests result cards’ |  |  |  |
| Not aware | 770 | 160 | 1 (reference) |
| Aware | 380 | 190 | 2.33 (1.77–3.07) |
|  | A correct answer for SQ7-3  (You are going to eat hot pot with someone who has hepatitis C. What is the probability that you will become infected by eating dishes on the same big pot with them?) | | Odds ratio (95% CI) ^a)^ |
| The word awareness of notification | No | Yes |  |
| Not aware | 635 | 238 | 1 (reference) |
| Aware | 418 | 209 | 1.5 (1.16–1.94) |
| The word awareness of ‘Negative hepatitis virus tests result cards’ |  |  |  |
| Not aware | 668 | 262 | 1 (reference) |
| Aware | 385 | 185 | 1.33 (1.03–1.73) |

a) Adjustment for occupation (human resource personnel/executives) and company size.

# Supplementary Table S4 Association between detailed knowledge of the notification or ‘Negative hepatitis virus tests result cards’ and testing for hepatitis in sensitivity analysis (Analysing the detailed knowledge of the notification or ‘Negative hepatitis virus tests result cards’ categories in Table 1).

|  | Testing for hepatitis | | Odds ratio (95% confidence interval) | | |
| --- | --- | --- | --- | --- | --- |
|  | No | Yes | Model 1 a) | Model 2 b) | Model 3 c) |
| The detailed knowledge of notification |  |  |  |  |  |
| Not knowledgeable | 728 | 582 | 1 (reference） | 1 (reference） | 1 (reference） |
| Knowledgeable | 6 | 184 | 38.4 (16.9–87.2) | 30.3 (13.2–69.2) | 26.6 (11.6–60.9) |
| The detailed knowledge of ‘Negative hepatitis virus tests result cards’ |  |  |  |  |  |
| Not knowledgeable | 728 | 590 | 1 (reference） | 1 (reference） | 1 (reference） |
| Knowledgeable | 6 | 176 | 36.2 (15.9–82.3) | 27.6 (12.1–63.1) | 24.4 (10.7–56.1) |

a) Unconditional logistic regression model.

b) Adjustment for occupation (human resources/executives) in Model 1.

c) Additional adjustment for company size in Model 2.

# Supplementary Table S5 Characteristics of participants and their companies in sensitivity analysis (population only excluded participants with inappropriate answers and unknown hepatitis test status) stratified by word awareness of the notification

|  |  | No word awareness of the notification | | Word awareness of the notification | |
| --- | --- | --- | --- | --- | --- |
| Population, no. |  | 1,045 | | 703 | |
| Male |  | 881 (84.3%) | | 538 (76.5%) | |
| Age, year |  | 54.8 (±9.6) | | 49.5 (±11.8) | |
| Company size (Number of employees) |  |  | |  | |
| Under 10 persons |  | 372 (35.6%) | | 74 (10.5%) | |
| 10 to 49 persons |  | 172 (16.5%) | | 72 (10.2%) | |
| 50 to 99 persons |  | 91 (8.7%) | | 74 (10.5%) | |
| 100 to 299 persons |  | 144 (13.8%) | | 122 (17.4%) | |
| 300 to 499 persons |  | 57 (5.5%) | | 73 (10.4%) | |
| 500 to 999 persons |  | 64 (6.1%) | | 74 (10.5%) | |
| 1000 persons or more |  | 144 (13.8%) | | 213 (30.3%) | |
| Unknown |  | 1 (0.1%) | | 1 (0.1%) | |
| Industry |  |  | |  | |
| Agriculture and forestry |  | 3 (0.3%) | | 4 (0.6%) | |
| Fisheries |  | 1 (0.1%) | | 2 (0.3%) | |
| Mining and quarrying of stone and gravel |  | 2 (0.2%) | | 5 (0.7%) | |
| Construction |  | 98 (9.4%) | | 52 (7.4%) | |
| Manufacturing |  | 176 (16.8%) | | 115 (16.4%) | |
| Electricity, gas, heat supply, and water |  | 22 (2.1%) | | 20 (2.8%) | |
| Information and communications |  | 112 (10.7%) | | 49 (7%) | |
| Transport and portal service |  | 45 (4.3%) | | 33 (4.7%) | |
| Wholesale and retail trade |  | 117 (11.2%) | | 57 (8.1%) | |
| Finance and insurance |  | 51 (4.9%) | | 54 (7.7%) | |
| Real estate, goods rental, and leasing |  | 91 (8.7%) | | 47 (6.7%) | |
| Scientific research, professional and technical services | 57 (5.5%) | | 23 (3.3%) | |  |
| Accommodations, eating and drinking services | 27 (2.6%) | | 16 (2.3%) | |  |
| Living-related and personal services and amusement services | 26 (2.5%) | | 22 (3.1%) | |  |
| Education, learning support |  | 22 (2.1%) | | 27 (3.8%) | |
| Medical, health care and welfare |  | 52 (5%) | | 75 (10.7%) | |
| Compound services |  | 6 (0.6%) | | 13 (1.8%) | |
| Service, N.E.C. |  | 137 (13.1%) | | 89 (12.7%) | |
| Government, except elsewhere classified |  | 0 (%) | | 0 (%) | |
| Position |  |  | |  | |
| Executives |  | 676 (64.7%) | | 204 (29%) | |
| Head of a department |  | 80 (7.7%) | | 153 (21.8%) | |
| Section chief |  | 84 (8%) | | 153 (21.8%) | |
| Managers |  | 67 (6.4%) | | 98 (13.9%) | |
| Staff |  | 138 (13.2%) | | 95 (13.5%) | |
| Health insurance association |  |  | |  | |
| In-house health insurance association |  | 179 (17.1%) | | 246 (35%) | |
| Parent company's health insurance association | 65 (6.2%) | | 84 (11.9%) | |  |
| Health insurance association in the industry |  | 140 (13.4%) | | 85 (12.1%) | |
| National Health Insurance Association (Kyokai kempo) |  | 542 (51.9%) | | 235 (33.4%) | |
| Mutual Aid Associations and Mutual Aid Programs | 21 (2%) | | 24 (3.4%) | |  |
| Seafarers’ Insurance |  | 0 (0%) | | 3 (0.5%) | |
| National Health Insurance |  | 72 (6.9%) | | 18 (2.6%) | |
| Other |  | 2 (0.2%) | | 1 (0.1%) | |
| Unknown |  | 24 (2.3%) | | 7 (1%) | |
| Occupation |  |  | |  | |
| Human resources |  | 369 (35.3%) | | 503 (71.6%) | |
| Executive |  | 676 (64.7%) | | 200 (28.4%) | |
| Employees with HBV or HCV infection |  |  | |  | |
| Unknown |  | 171 (16.4%) | | 75 (10.7%) | |
| Yes |  | 55 (5.3%) | | 258 (36.7%) | |
| No |  | 819 (78.4%) | | 370 (52.6%) | |
| Hepatitis testing for particular employees |  |  | |  | |
| Yes |  | 282 (27%) | | 568 (80.8%) | |
| No |  | 763 (73%) | | 135 (19.2%) | |

Percentage may not add up to 100 because of rounding.

# Supplementary Table S6 Association between word awareness of the notification or ‘Negative hepatitis virus tests result cards’ and testing for hepatitis in sensitivity analysis (population only excluded participants with inappropriate answers and unknown hepatitis test status)

|  | Testing for hepatitis | | Odds ratio (95% confidence interval) | | |
| --- | --- | --- | --- | --- | --- |
|  | No | Yes | Model 1 a) | Model 2 b) | Model 3 c) |
| The word awareness of notification |  |  |  |  |  |
| Not aware | 763 | 282 | 1 (reference） | 1 (reference） | 1 (reference） |
| Aware | 135 | 568 | 11.4 (9.02–14.4) | 9.77 (7.68–12.4) | 9.44 (7.41–12) |
| The word awareness of ‘Negative hepatitis virus tests result cards’ |  |  |  |  |  |
| Not aware | 799 | 326 | 1 (reference） | 1 (reference） | 1 (reference） |
| Aware | 99 | 524 | 13 (10.1–16.7) | 11.1 (8.57–14.3) | 10.7 (8.28–13.9) |

a) Unconditional logistic regression model.

b) Adjustment for occupation (human resources/executives) in Model 1.

c) Additional adjustment for company size in Model 2.

# Supplementary Table S7 Comparison of hepatitis quizzes

| This study’s quizzes question | Answers  (correct answer) | Previous study’s quizzes for general populations^a)^ | Answers ^a)^  (correct answer) |
| --- | --- | --- | --- |
| SQ7-1 Among the items currently included in regular health checkups, viral hepatitis can be detected. | 1) If it's covered in a routine health checkup, it can be detected nearly 100% of the time.  2) About half (around 50%) of the items covered in a routine health checkup can be detected.  3) It can hardly ever be detected in a routine health checkup.  4) It's unclear whether it can be detected in a routine health checkup.  (correct answer: 3) | Even if liver function tests are normal during a health checkup, could I still be infected with a hepatitis virus or have fatty liver disease? | Yes /No  (correct Answer: Yes) |
| SQ7-2 Current HCV infection can be treated. | 1) Most (over 95%) can be treated with oral medication. 2) Most (over 95%) can be treated with injectable medication (interferon). 3) Some people undergo treatment with oral medication. 4) Some people undergo treatment with injectable medication (interferon). 5) It is unclear whether treatment is possible.   (correct answer: 1) | What are the main treatments for hepatitis B and hepatitis C? | Oral medication / hospitalisation with IV drip  (correct Answer: Oral medication) |
| SQ7-3 You are going to eat hot pot with someone who has hepatitis C. What is the probability that you will become infected by eating dishes on the same big pot with them? | 1) 0% 2) Around 2% 3) Around 20% 4) Over 80% 5) Don't know   (correct answer: 1) | Can’t hepatitis B or C viruses be transmitted by mosquitoes (or through eating hot pots or baths)? | Yes / No  (Correct Answer: No ^b)^) |

a) Masuda, S., et al., Knowledge Enlightenment About Viral Hepatitis Using a Quiz at an Awareness-Raising Event. Kanzo, 2025. 66(10): p. 443-446.

b) In original transcription, the correct answer in Japanese is Yes (Hepatitis B or C virus cannot be transmitted).
